# Fever lasting 48 hours as a predictive factor of ESBL-producing bacteria in non-critically ill patients with urinary tract infection

**DOI:** 10.1038/s41598-024-61824-7

**Published:** 2024-05-13

**Authors:** Sungbin Yoon, Hae-rim Kim, So Won Kim, Hoon Yu

**Affiliations:** 1 Division of Nephrology, Department of Internal Medicine, Gangneung Asan Hospital, University of Ulsan College of Medicine, Gangneung, Republic of Korea; 2 School of Statistics, College of Natural Science, Seoul National University, Seoul, Republic of Korea; 3 Department of Pharmacology, Asan Medical Center, University of Ulsan College of Medicine, 88, Olympic-ro 43-gil, Songpa-gu, Seoul, 05505 Republic of Korea; 4 Department of Nephrology, Gangneung Asan Hospital, University of Ulsan College of Medicine, Bangdong-gil 38, Sacheon-myeon, Gangneung-si, Gangwon-do 25440 Republic of Korea

**Keywords:** Prolonged fever, Predictive factor, Extended spectrum β-lactamase inhibitor, *Escherichia coli*, Urinary tract infection, Acute pyelonephritis, Diseases, Nephrology, Risk factors, Signs and symptoms, Urology

## Abstract

Urinary tract infection (UTI) is the most prevalent urological condition worldwide. Choosing appropriate antibiotics for patients who have fever before receiving a culture result is challenging. This retrospective study enrolled patients 394 patients hospitalized at Gangneung Asan Hospital for UTI from May 2017 to April 2021. Fever at 48 h of hospitalization was the analysis point, as this is when the response to antibiotic therapy manifest, although the results of antibiogram are not available. Multivariate analysis was performed to assess the correlation between ESBL producing bacteria (EPB) and fever at 48 h. Overall, 36.3% of patients had EPB and 27.9% had fever at 48 h. In multivariate analysis, a significant positive association was found between EPB and fever (odds ratio 1.17, 95% CI 1.05–1.30, *P* = 0.004) Female had negative association with multivariate model (OR 0.83, 95% CI 0.73–0.94, *P* = 0.004). Diabetes did not demonstrate a significant association with EPB. (OR 1.10, 95% CI 0.99–1.22, *P* = 0.072). Fever at 48 h is associated with EPB and could be considered a predictive factor for EPB infection in patients with UTI. Antibiotic escalation may be considered in patients with fever at 48 h.

## Introduction

Urinary tract infection (UTI) ranks among the most prevalent conditions encountered in urological practice. Clinical protocols generally indicate the initiation of antibiotic therapy pre-emptively in patients with UTI, without awaiting urine culture and sensitivity results, with subsequent adjustments to the antibiotic regimen made as necessary^1^. However, the current landscape of escalating antibiotic resistance has led to a noticeable delay in the therapeutic efficacy of empirical antibiotic regimens for UTI^2^. The 2021 antimicrobial resistance report published by the World Health Organization highlights this pressing issue, showing that *E. coli*, a primary UTI pathogen, exhibits a resistance rate to ciprofloxacin of 43.1% (interquartile range [IQR] 22.5–58.6) and a 33–46% resistance rate to third generation cephalosporins. Similarly, *K. pneumoniae* shows a ciprofloxacin resistance rate of 36.4% (IQR 28.5–52.3) and a 44–51% resistance to third-generation cephalosporins^3^. These results highlight the urgency for the medical community to prioritize the prediction of antibiotic-resistant bacterial strains and administration of targeted antibiotic treatments in advance of the availability of culture and sensitivity results.

In clinical settings, empirical antibiotic therapy for UTI is initiated rapidly following admission, which is typically followed by a waiting period of 3–5 days before the results of bacterial culture and antimicrobial susceptibility testing can be confirmed. In response to this delay, research efforts to develop predictive models for antibiotic-resistant bacterial infections in UTI have intensified. Emerging evidence from various studies has indicated that certain clinical factors are strongly correlated with the risk of antibiotic-resistant infections. These factors include advanced age, male sex, history of UTIs within the past year, antibiotic treatment within the previous 3 months, and the presence of diabetes mellitus^4–7^ are particularly relevant prior to the initiation of treatment.

Currently, no predictive indicators of antibiotic resistance have been identified that can be used following the commencement of empirical antibiotic therapy and before the availability of drug susceptibility test results. However, fever is recognized as a critical clinical marker in the context of antibiotic stewardship, particularly for its immediacy in signaling the response to treatment. The manifestation of fever in clinical infections can serve as an acute indicator of the efficacy of the administered treatment, particularly through its pattern of resolution. Typically in UTI, an appropriate treatment regimen results in the presence of fever for approximately 2–3 days, with a noticeable decline in fever usually initiating by the fourth day^8^.

Conversely, the treatment response to antibiotics can be presumed to be suboptimal in cases of UTI caused by antibiotic-resistant bacteria. This is commonly evidenced by an extended duration and increased intensity of fever compared to infections caused by nonresistant bacteria. Consequently, the persistence of fever beyond the expected timeframe may be indicative of failure of the empirical antibiotic treatment. Thus, recommendation to alter the antibiotic regimen may be needed. This approach underscores the need for vigilant monitoring of fever as a parameter for adjusting treatment protocols according to the management of UTI^9^.

We aimed to determine the potential of prolonged fever as a predictive tool for the presence of resistant bacterial strains, which could significantly influence the decision-making process in the empirical treatment of UTIs, thereby promoting the judicious use of antibiotics and optimizing patient outcomes.

## Results

**Figure 1:**
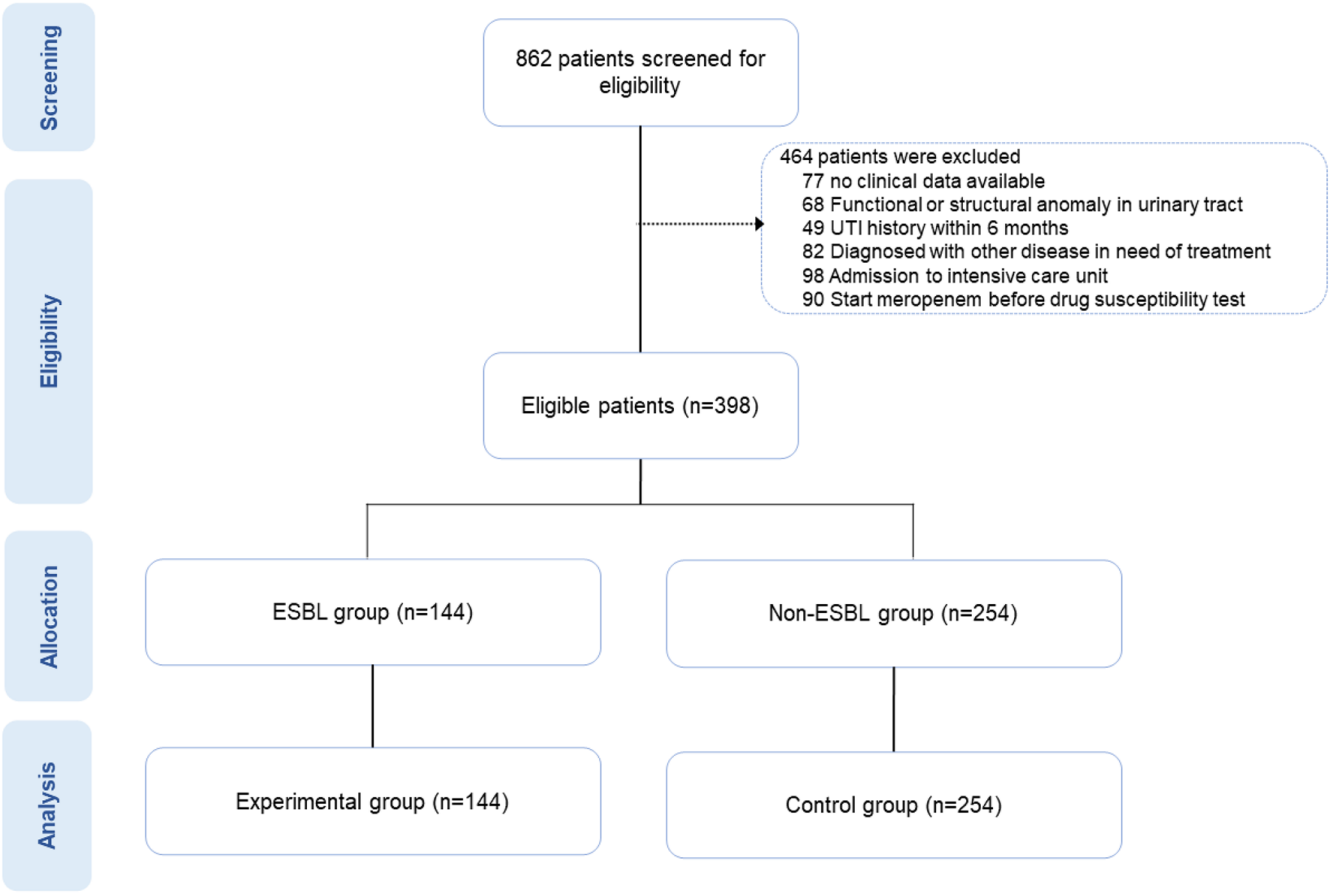
Study flowchart. Overall, 862 patients were screened, of whom 468 patients were excluded based on the exclusions criteria. Finally, 394 patients were deemed eligible for the study. Patients were divided into two groups based on whether or not they had ESBL.

Figure 1 presents a flow chart of the study design. Of the 862 patients initially considered for the study, 464 were excluded based on the predetermined exclusion criteria. The reasons for exclusion were as follows: absence of essential clinical data (77 patients), presence of functional or structural anomalies within the urinary tract (68 patients), a history of UTI within the past 6 months (49 patients), a diagnosis requiring treatment for other diseases (82 patients), necessity for admission to the intensive care unit (98 patients), and the initiation of meropenem therapy before the availability of drug susceptibility test results (90 patients) After applying these criteria, 398 patients were deemed eligible for inclusion in the study, and were subsequently categorized into two groups based on the results of their drug susceptibility tests: 144 patients were identified with infections caused by ESBL-producing bacteria (ESBL group), while 254 patients had infections caused by ESBL-nonproducing bacteria (non-ESBL group).

**Figure 2:**
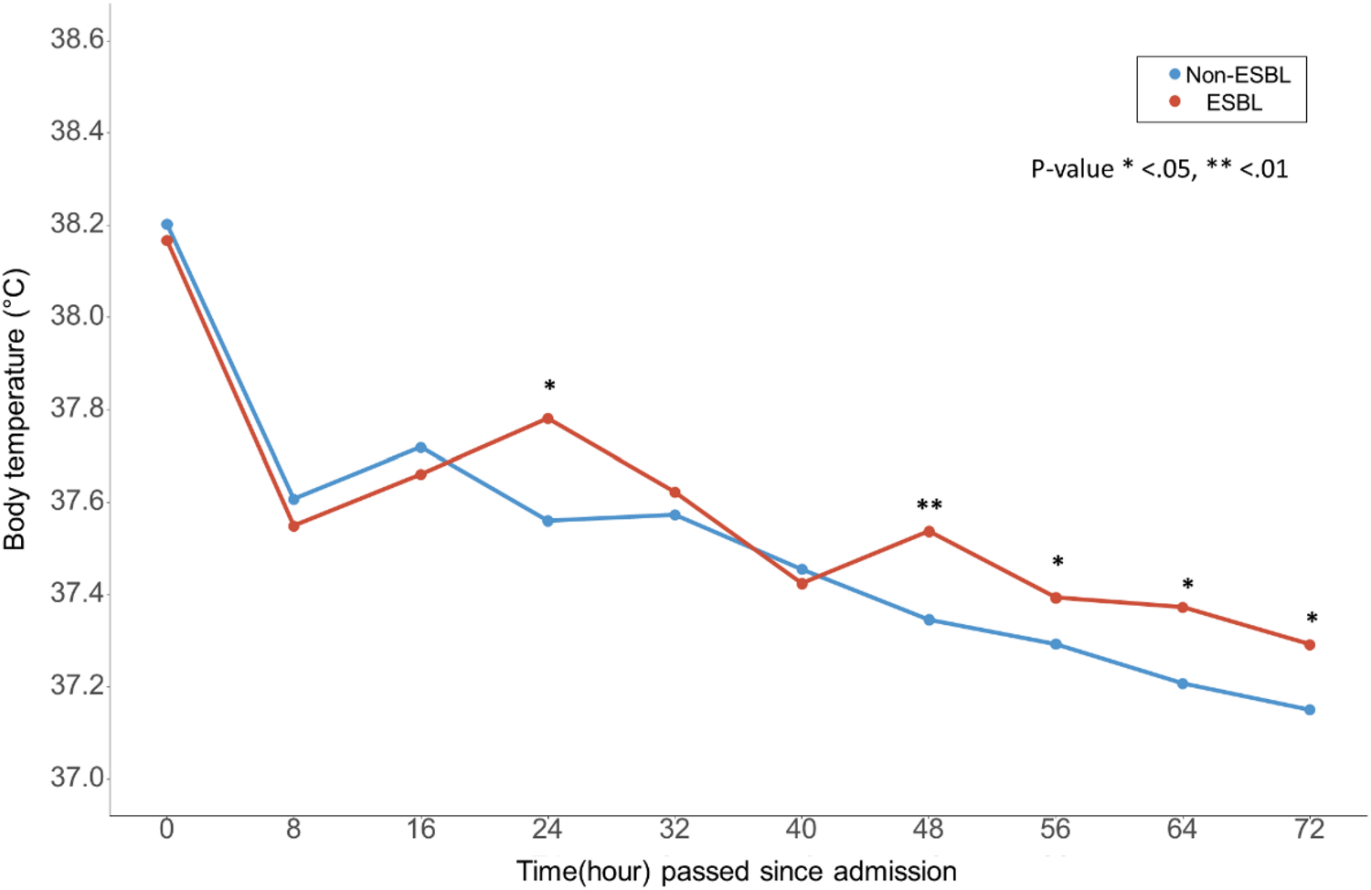
Fever change over time between the two groups. Graphs showing the average fever in each group, plotted over time. Fever at 24 and 48 h showed statistical differences between the ESBL positive group and ESBL negative group.

**Figure 3:**
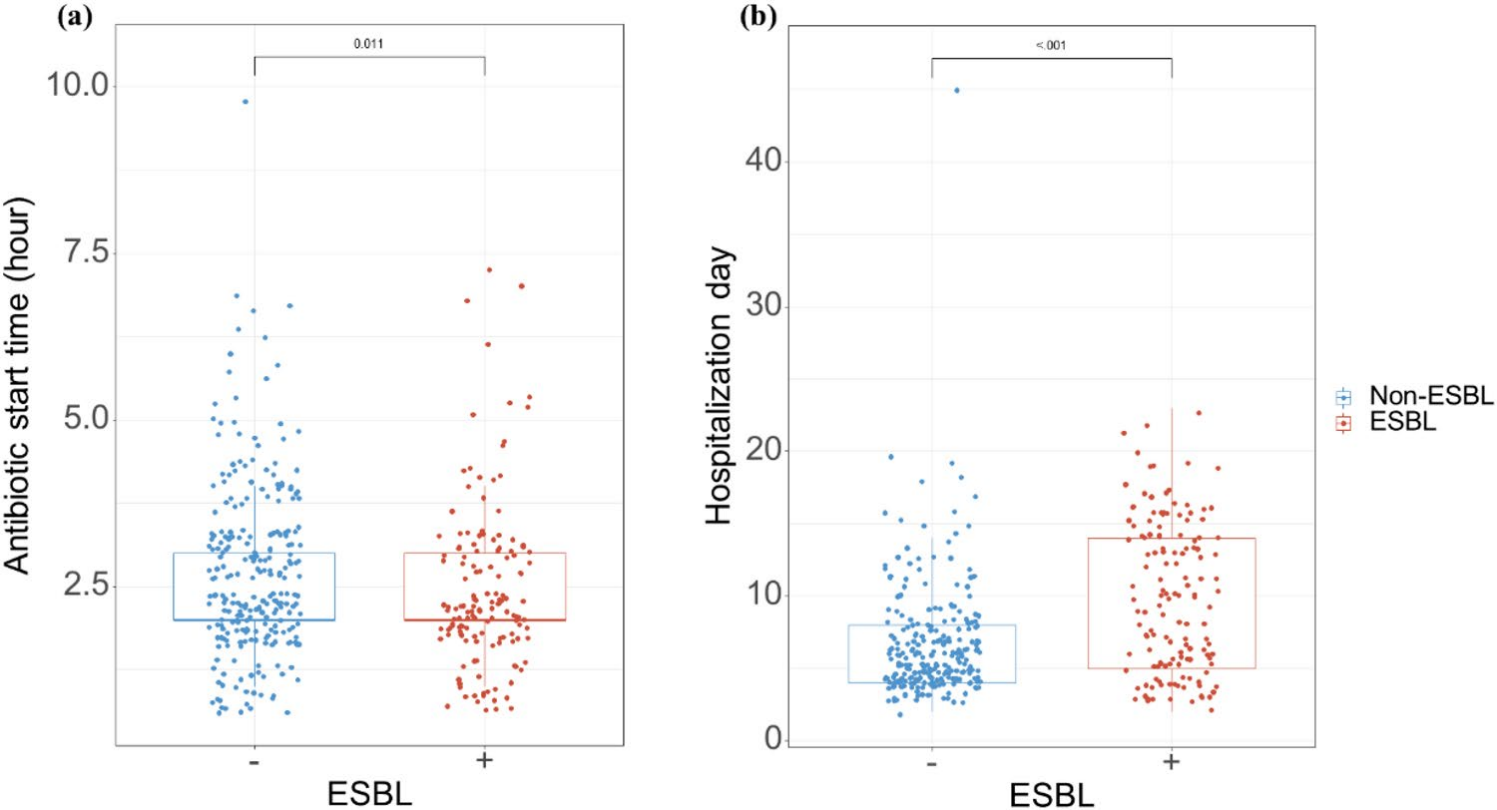
Pivotal plots for antibiotic start time and hospital days between the two groups. Antibiotics start time (**a**) and hospitalization day (**b**) of all patients between the ESBL positive negative groups.

Table 1 shows the baseline characteristics of the study population. The majority of the patients were females (82.41% of the total), with females accounting for 75.69 and 86.22% of the ESBL and non-ESBL groups, respectively. The mean age of the entire group was 70.84 ± 14.12 years. Regarding body mass index, the overall mean was 24.29 ± 4.44, with the ESBL group averaging 23.95 ± 4.61 and the non-ESBL group 24.48 ± 4.34. Diabetes prevalence was 31.91% across all patients, with a higher incidence in the ESBL group (38.19%) than that in the non-ESBL group (28.35%), although this difference did not reach statistical significance (*P* = 0.056). Chronic kidney disease (CKD) of any stage was present in 6.53, 7.64, and 5.91% of all patients and ESBL and non-ESBL groups, respectively (*P* = 0.645). Hospital stay duration varied significantly between groups; the ESBL group had a mean hospitalization of 9.90 ± 5.19 days, compared to 6.69 ± 4.05 days in the non-ESBL group (*P* < 0.001). The initiation of antibiotic treatment occurred sooner in the ESBL group (2.44 ± 1.22 days) than in the non-ESBL group (2.74 ± 1.31 days). Over 95% of patients received either cephalosporin or fluoroquinolone as their initial antibiotic therapy. No notable disparities were observed in CT findings across the groups. However, fever patterns differed; at 48 h postadmission, the ESBL group had a higher average body temperature (37.54 ± 0.80 °C) compared to the non-ESBL group (37.35 ± 0.64 °C, *P* = 0.015). Furthermore, a body temperature exceeding 37.7 °C at 48 h was more common in the ESBL group (36.11%) than in the non-ESBL group (23.32%). Figure 2 provides a graphical depiction of the variation in fever over time between the two patient groups. Overall, we observed that the ESBL group exhibited a marginally higher fever than the non-ESBL group, which was particularly noticeable from 24 h postadmission. Our study showed pivotal plot for antibiotic start time and hospital day between two groups in Fig. 3.

**Table 1:** Baseline characteristics of the study participants.

	Total	ESBL	Non-ESBL	*P*-value
	(N = 398)	(N = 144)	(N = 254)	
Female sex	328 (82.41%)	109 (75.69%)	219 (86.22%)	0.012
Age, years	70.84 ± 14.12	71.54 ± 14.06	70.44 ± 14.16	0.457
Body mass index, kg/m^2^	24.29 ± 4.44	23.95 ± 4.61	24.48 ± 4.34	0.258
Hypertension	231 (58.04%)	79 (54.86%)	152 (59.84%)	0.389
Diabetes	127 (31.91%)	55 (38.19%)	72 (28.35%)	0.056
Liver cirrhosis	4 (1.01%)	1 (0.69%)	3 (1.18%)	1.000
Heart failure	22 (5.53%)	10 (6.94%)	12 (4.72%)	0.482
Chronic kidney disease	26 (6.53%)	11 (7.64%)	15 (5.91%)	0.645
Hospitalization, days	7.85 ± 4.75	9.90 ± 5.19	6.69 ± 4.05	< .001
Antibiotic start time, hours	2.63 ± 1.29	2.44 ± 1.22	2.74 ± 1.31	0.027
Initial antibiotic				0.929
Cephalosporin	194 (48.74%)	68 (47.22%)	126 (49.61%)	
Fluoroquinolone	195 (48.99%)	72 (50.00%)	123 (48.43%)	
P/β	7 (1.76%)	3 (2.08%)	4 (1.57%)	
Others	2 (0.50%)	1 (0.69%)	1 (0.39%)	
Hemoglobin, g/dL	12.02 ± 1.66	11.93 ± 1.66	12.06 ± 1.66	0.457
WBC counts, × 10^3^/uL	13.35 ± 5.24	13.05 ± 5.21	13.51 ± 5.26	0.405
C-reactive protein, mg/dL	16.56 ± 9.82	15.04 ± 8.14	17.43 ± 10.58	0.015
Serum albumin, g/dL	3.59 ± 0.43	3.60 ± 0.41	3.58 ± 0.45	0.695
CT finding				0.956
Negative	122 (30.65%)	43 (29.86%)	79 (31.10%)	
Unilateral	176 (44.22%)	65 (45.14%)	111 (43.70%)	
Bilateral	100 (25.13%)	36 (25.00%)	64 (25.20%)	
Organism				0.748
*E. coli*	394 (98.99%)	143 (99.31%)	251 (98.82%)	
*K. Oxytoca*	3 (0.75%)	1 (0.69%)	2 (0.79%)	
*K. Pneumoniae*	1 (0.25%)	0 ( 0.0%)	1 (0.39%)	
Fever at 48 h	37.41 ± 0.71	37.54 ± 0.80	37.35 ± 0.64	0.015
over 37.2 °C at 48 h	227 (57.18%)	93 (64.58%)	134 (52.96%)	0.032
over 37.4 °C at 48 h	172 (43.32%)	76 (52.78%)	96 (37.94%)	0.006
over 37.7 °C at 48 h	111 (27.96%)	52 (36.11%)	59 (23.32%)	0.009
over 37.9 °C at 48 h	84 (21.16%)	42 (29.17%)	42 (16.60%)	0.005

Continuous variables are presented as the mean ± standard deviation and categorical variables are presented as number (percentage).

CT, computed tomography; P/β, penicillin/β-lactamase inhibitor; WBC, white blood cell; *E. coli*, *Eschericia coli*; *K. oxytoca*, *Klebsiella oxytoca*; *K. pneumoniae*, *Klebsiella pneumoniae*.

The findings from both univariate and multivariate logistic regression analyses exploring the relationship between prolonged fever and ESBL producing bacteria (EPB) are presented in Table 2. The univariate analysis indicated a positive association between prolonged fever at 48 h and the presence of EPB (odds ratio [OR] 1.16, 95% confidence interval [CI] 1.04–1.29, *P* = 0.006). This association remained consistent in both multivariate models: model 1 (OR 1.16, 95% CI 1.05–1.29, *P* = 0.005) and model 2 (OR 1.17, 95% CI 1.05–1.30, *P* = 0.004). Female gender exhibited a negative association with EPB in the univariate analysis (OR 0.85, 95% CI 0.75–0.96, *P* = 0.008), a finding which was confirmed in both multivariate models: model 1 (OR 0.83, 95% CI 0.73–0.94, *P* = 0.004) and model 2 (OR 0.84, 95% CI 0.74–0.95, *P* = 0.005). Early initiation of antibiotic therapy showed an association with EPB across both univariate and multivariate analyses (univariate OR 0.96, 95% CI 0.92–1.00, *P* = 0.027; multivariate model 1 OR 0.96, 95% CI 0.93–1.00, *P* = 0.039; model 2 OR 0.96, 95% CI 0.93–1.00, *P* = 0.044). Diabetes, however, did not demonstrate a significant association with EPB in multivariable analyses (multivariate model 1 OR 1.10, 95% CI 1.00–1.22, *P* = 0.059; model 2 OR 1.11, 95% CI 1.00–1.23, *P* = 0.052). While CRP levels appeared to be statistically associated with EPB, they were deemed not to be a reliable marker because of the poor OR (0.99 in the univariate and multivariate model 1,2, respectively).

**Table 2:** Logistic regression model assessing the link between prolonged fever and ESBL-producing bacteria.

	Univariate		Model 1		Model 2	
	OR (95% CI)	*P*	OR (95% CI)	*P*	OR (95% CI)	*P*
Fever at 48 h	1.16 (1.04–1.29)	0.006	1.16 (1.05–1.29)	0.005	1.17 (1.05–1.30)	0.004
Sex (female)	0.85 (0.75–0.96)	0.008	0.83 (0.73–0.94)	0.004	0.84 (0.74–0.95)	0.005
Age	1.00 (1.00–1.00)	0.457	1.00 (1.00–1.00)	0.422	1.00 (1.00–1.00)	0.518
BMI	0.99 (0.98–1.00)	0.258			0.99 (0.98–1.00)	0.226
DM	1.11 (1.00–1.23)	0.043	1.10 (1.00–1.22)	0.059	1.11 (1.00–1.23)	0.052
CKD	1.07 (0.88–1.29)	0.503			1.01 (0.83–1.23)	0.892
HTN	0.95 (0.87–1.05)	0.334				
LC	0.89 (0.56–1.44)	0.641				
HF	1.10 (0.90–1.36)	0.353				
Antibiotic start time	0.96 (0.92–1.00)	0.027	0.96 (0.93–1.00)	0.039	0.96 (0.93–1.00)	0.044
Initial antibiotic						
Cephalosporin	Ref					
Fluoroquinolone	1.02 (0.93–1.12)	0.702				
P/β	1.08 (0.75–1.56)	0.674				
Others	1.16 (0.59–2.27)	0.663				
Hemoglobin	0.99 (0.96–1.02)	0.457				
WBC count	1.00 (0.99–1.01)	0.405				
CRP	0.99 (0.99–1.00)	0.023	0.99 (0.99–1.00)	0.021	0.99 (0.99–1.00)	0.025
Albumin	1.02 (0.92–1.14)	0.695				

Model 1: sex, antibiotic start, CRP, DM, age.

Model 2: sex, antibiotic start, CRP, DM, age, BMI, CKD.

Logistic regression model for prolonged fever. Adjustable items were selected by univariate model and clinically importance.

BMI, body mass index; CKD, chronic kidney disease; CT, computed tomography; DM, diabetes mellitus; HF, heart failure; HTN, hypertension; LC, liver cirrhosis; P/β, penicillin/β-lactamase inhibitor; WBC, white blood cell.

## Discussion

Overall, our study showed that fever at 48 h was strongly associated with the presence of ESBL-producing bacteria in newly diagnosed, non-critically ill UTI patients. Even when we adjusted for other known risk factors (advanced age, the presence of diabetes mellitus) of antibiotic-resistant infections, prolonged fever was still strongly associated with ESBL producing bacteria.

The findings of our research indicated that 36.2% of bacteria isolates were producers of ESBL. Among these EPB, a high percentage (90.9%) demonstrated resistance to cephalosporin-class antibiotics. Additionally, a significant proportion (66.4%) were resistant to fluoroquinolones. These resistance patterns closely mirror those observed in national surveillance data. For example, antibiogram data from urinary cultures in Korea spanning the years 2018–2020 revealed considerable resistance among the isolated bacteria^10^.

In our investigation, we observed a predominant initial prescription pattern for UTIs that favored fluoroquinolones (48.99%) and cephalosporins (48.74%), which is in agreement with current clinical protocols. The preference for fluoroquinolones in South Korea is attributed to the elevated resistance rates to co-trimoxazole. Nevertheless, considerable resistance to fluoroquinolones exists among UTI pathogens in Korea. Consequently, intravenous cephalosporins have arisen as a common therapeutic recourse for UTIs within the region^2^.

In our study, we observed that among the non-ESBL group, 22/254 patients were initially treated with fluoroquinolone as the empirical antibiotic, despite subsequent culture drug susceptibility tests indicating resistance to fluoroquinolone. Furthermore, only one patient was initially treated with cephalosporin as the empirical antibiotic, despite subsequent culture drug susceptibility tests indicating resistance to cephalosporin. Interestingly, the fever patterns at 48 h post administration in fluoroquinolone-resistant patients did not significantly differ from fluoroquinolone-susceptible patients in the ESBL negative group (Supplementary Table 1). This suggests that even in cases where the causative microorganism is resistant to the empirical antibiotic, the presence of ESBL positivity may be a more significant factor in the manifestation of high fever at the 48-h mark.

Investigation of the primary outcome of our study revealed a significant association between fever at 48 h and the presence of ESBL-producing bacteria. This result indicates that both the duration and intensity of fever are linked to the existence of antibiotic-resistant organisms. Specifically, an extended period of fever could be indicative of the ineffectiveness of empirical antibiotic therapy. A prior European prospective observational study investigating pediatric patients with fever lasting 5 days or more concluded that prolonged fever is associated with a higher risk of serious illness^11^. Under normal circumstances, successful empirical antibiotic treatment should lead to a reduction in fever as the bacterial infection is resolved^12^. However, the intricacies of fever height in relation to antibiotic-resistant organisms remain somewhat ambiguous. Insights from a prior study conducted in the United States on infants with invasive bacterial infections indicated that more severe infections (such as meningitis) were associated with higher fevers compared to less severe infections (such as bacteremia)^13^. This pediatric study provides a hint that increased bacterial activity may correlate with higher fever. In other studies, the association between prolonged fever and severe bacterial infection was evident in adult patients presenting with high fever. However, the relationship between the height of the fever and the severity of the bacterial infection has not been as clearly defined^14,15^. Prolonged fever can be a strong indicator of a serious infection in adults, the peak temperature reached does not necessarily correlate directly with the severity of the infection.

Typically, the treatment duration for urinary tract infections ranges 5–10 days, and depends on a variety of clinical factors, including negative culture results, improvements in laboratory parameters, and resolution of symptoms. Importantly, our study highlights that patient in the ESBL group experienced longer hospital stays. Despite the initiation of antibiotic therapy being swifter in the ESBL group compared to the non-ESBL group, we observed a noticeable prolongation in their hospitalization duration. Additionally, a higher proportion of patients in the ESBL group presented with elevated fever after 48 h of admission compared to the non-ESBL group. These findings suggest that delays in administering the appropriate antibiotic can lead to prolonged fever episodes. Consequently, such prolonged fever can hinder clinical recovery, thereby extending the duration of hospitalization and delaying patient discharge. This outcome emphasizes the critical importance of timely and effective antibiotic therapy in managing UTIs, particularly in the context of antibiotic resistance (Fig. 3).

Several studies have indicated that certain factors are strongly correlated with the risk of antibiotic-resistant infections. These factors include advanced age, male gender, history of UTIs within the past year, antibiotic treatment within the previous 3 months, and the presence of diabetes mellitus^4–7^. Unfortunately, these identified factors are particularly relevant prior to the initiation of treatment and do not affect antibiotic choice. Overall, our study aimed to evaluate prolonged fever during empirical treatment as a predictive factor for escalation of antibiotics. As such, patients with a history of UTIs within the previous 6 months, or those who required of intensive care unit admission was excluded. This is because patients with sepsis or septic are recommended to take two broad-spectrum antibiotics or carbapenem as empirical antibiotics, due to the poor clinical outcomes^16,17^.

In our study, female sex was negatively correlated with the ESBL group, which is in agreement with existing studies. However, we found no significant association between diabetes and the ESBL group. This finding diverges from some studies which previously identified risk factors^4–7^. This discrepancy may stem from the due to variations in the study populations. We focused on a cohort that primarily consisted of patients with non-critically ill UTIs. Additionally, our study population only included patients who had not been administered antibiotics for more than 6 months prior to the study. This contrasts with other studies, which generally included a broader range of patients, including those with more complex medical histories and recent antibiotic usage.

Analysis of the laboratory data collected at the treatment onset (day 0), encompassing CRP levels, WBC count, and hemoglobin and albumin levels, revealed no significant disparities between the two groups in our study. However, due to our clinical policy to sublate habitual laboratory test in non-critically ill UTIs and the retrospective nature of the study design, we only had access to 65% of comprehensive laboratory data at the 2-d hospitalization mark. This limitation hindered our ability to assess the trajectory of laboratory recovery for the enrolled patients.

This retrospective analysis used an infrared tympanic thermometer to assess the body temperature of patients. The convenience of the device contributed to its widespread use in our facility. Currently, according to Harrison’s Principles of Internal Medicine, fever is defined as a rectal temperature of 37.5–38.3 °C (99.5°F–100.9°F) with lower thresholds applicable to frail elderly persons. The Merck Manual defines fever as an oral temperature > 37.8 °C (> 100.0°F) or a rectal temperature > 38.2 °C (> 100.8°F) or a temperature higher than a person’s known normal daily value. Several studies have noted that the infrared tympanic thermometer tends to record temperatures that are, on average, 0.5 °C lower compared to other measurement tools^18,19^. In addition, we provided a restricted odds ratio spline curve for fever at 48 in Supplementary Fig. 1. This curve indicates that body temperature increase correlates with a higher odds ratio for ESBL-producing bacterial presence. Furthermore, Supplementary Tables 2 and 3 present logistic regression models for body temperatures of 37.4 °C and 37.9 °C. These tables also confirm that the odds ratio for ESBL-producing bacterial presence increases in parallel with rising body temperatures. Therefore, we selected a temperature beyond 37.7 °C as a threshold value to define fever.

Our study possesses several positive aspects. Firstly, it reinforces the notion that prolonged fever can serve as a key factor in deciding to escalate antibiotic treatment prior to culture results, particularly in patients with non-critically ill UTIs. Secondly, we used a convenient tool—the infrared tympanic thermometer—to aid in the formulation of appropriate antibiotic strategies. However, this study also has several notable limitations. Firstly, it was conducted as a single-center retrospective analysis, there were no clear indication for admission. Which may limit the generalizability of the findings. Therefore, larger-scale, multi-center, or prospective studies are warranted to validate these findings for broader clinical application. Secondly, there is currently no standardized protocol for measuring body temperature using an infrared tympanic thermometer. Without a uniform approach, the recorded body temperatures may vary depending on the attending nurse who conducted the measurement. This variability could introduce inconsistencies in the temperature data, potentially affecting the reliability and accuracy of our findings regarding fever patterns in the patient groups.

## Conclusion

Prolonged fever is associated with EPB identification and could be considered as a predictive factor for infection caused by EPB in the treatment of UTI patients. Overall, our results showed that antibiotics escalation may be considered in patients with prolonged fever at 48 h post-treatment to achieve better clinical outcome.

## Methods

### Study population and design

This retrospective analysis enrolled a cohort of 862 adult (> 18 years) patients admitted and treated for UTI at Gangneung Asan Hospital from May 2017 to April 2021. The Institutional Review Board (IRB) of the Gangneung Asan hospital approved this research plan, which was constructed in compliance with Declaration of Helsinki (IRB number: GNAH 2023-11-015), and further waived the requirement for informed consent because of the deidentified data collection and retrospective nature of the study.

All participants had confirmed infections in either the urine, serum, or both. No participants changed antibiotics until the drug susceptibility test results were available. The selection of patients for inclusion was guided by specific exclusion criteria, which were as follows: lack of available clinical data (missing fever record, absence of abdominal computed tomography (CT) scan, and lack of laboratory data at admission), presence of functional or anatomical abnormalities in the urinary tract, history of UTI within the previous 6 months, concurrent diagnosis requiring treatment for another disease, requirement of intensive care unit admission, and the initiation of meropenem or ertapenem therapy prior to receiving drug susceptibility test results. Following the application of these criteria, a total of 394 adult patients were included in the final analysis.

The patient cohort was stratified into two groups based on the results of the drug susceptibility tests: the first comprised patients with infections caused by extended-spectrum beta-lactamase (ESBL)-producing bacteria (EPB), and the other comprised patients with infections caused by ESBL-negative bacteria. We meticulously collected and recorded data of body temperature (in °C), from the onset of hospitalization (day 0) through to day 5 of admission. Specific time points for temperature collection were at onset (hour 0) and subsequently at 8-h intervals for the first 72 h, followed by additional recording on day 4 and day 5. Fever was measured using infrared tympanic thermometer by attending nurse. In this study, fever at 48 h after admission (defined as body temperature above 37.7 °C) was chosen as the point of analysis.

### Data collection

Data for this investigation was extracted from the electronic medical records at Gangneung Asan Hospital. An array of demographic and clinical parameters was assembled, encompassing gender, age, height, body weight, length of hospital stays, and findings from abdominal CT scans. Additional clinical data collected included microbiological culture results, outcomes of drug susceptibility tests, the regimen of empirical antibiotic therapy, the number of days the patient was ill before hospital admission, and the presence of flank pain.

Patient medical histories were thoroughly reviewed, noting the presence of conditions such as hypertension, diabetes, chronic kidney disease, liver cirrhosis, heart failure, and any form of malignancy. Past instances of UTIs were also recorded. Fever was measured using an infrared tympanic thermometer. Fever progression was tracked, alongside a suite of pertinent laboratory markers, including white blood cell (WBC) and platelet counts and C-reactive protein (CRP) and serum albumin levels.

### Statistical analysis

Continuous variables are presented as the mean and standard deviation or median (interquartile range), while categorical variables are presented as frequency (percentage). Continuous variables were analyzed using a student’s *t*-test or Mann–Whitney test, depending on normality, and categorical variables were analyzed using a chi-square test or Fisher’s exact test and ANOVA for group comparisons. Univariable with logistic regression was performed to assess the correlation between EPB and fever at 48 h. To provide complementary analyses, we constructed a multivariate adjusted logistic regression model adjusted for patients’ baseline characteristics (as selected by the univariate model) and clinically important variables. Two-sided *P*-values of < 0.05 were considered significant. All statistical analyses and visualizations were conducted using R version 4.3.1 (The R Foundation, www.R-project.org) and IBM SPSS Statistics for Windows, version 27.0 (IBM Corp., Armonk, NY).

### Ethical approval

The Institutional Review Board (IRB) of the Gangneung Asan hospital approved the study protocol and waiving the requirement for informed consent because of deidentified data collection and retrospective nature of the study, which was constructed in compliance with the Declaration of Helsinki (IRB number: GNAH 2023–11-015).

### Supplementary Information

Supplementary Information.

## Data Availability

The datasets used and/or analysed during the current study are available from the corresponding author(HY) on reasonable request.

## Abbreviations

BMI: Body mass index
CI: Confidence interval
CKD: Chronic kidney disease
CT: Computed tomography
DM: Diabetes mellitus
*E. coli*: *Escherichia coli*
EPB: Extended-spectrum beta-lactamase producing bacteria
ESBL: Extended-spectrum beta-lactamase
FQ: Fluoroquinolone
HF: Heart failure
HTN: Hypertension
IRB: Institutional review board
IQR: Interquartile range
*K. Oxytoca*: *Klebsiella oxytoca*
*K. pneumoniae*: *Klebsiella pneumoniae*
LC: Liver cirrhosis
P/β: Penicillin/β-lactamase inhibitor
OR: Odds ratio
UTI: Urinary tract infection
WBC: White blood cell
