# Supplementary material for: Fever lasting 48 hours as a predictive factor of ESBL-producing bacteria in non-critically ill patients with urinary tract infection
Source: Sci Rep. 2024 May 13;14:10897. doi: 10.1038/s41598-024-61824-7 (PMC11091047; doi:10.1038/s41598-024-61824-7)
Supplement: Supplementary file 1 — Supplementary Information. [file 41598_2024_61824_MOESM1_ESM.docx]

**Supplementary Table/Figure**

**Supplementary Table 1. Baseline characteristics of the ESBL negative group administered fluoroquinolone as an empirical antibiotic**

|  | **Total** | **FQ resistance** | **FQ susceptible** | **p** |
| --- | --- | --- | --- | --- |
|  | **(N=126)** | **(N=22)** | **(N=104)** |  |
| Female sex | 107 (84.92%) | 19 (86.36%) | 88 (84.62%) | 1.000 |
| Age, years | 69.82 ± 14.30 | 70.50 ± 14.95 | 69.67 ± 14.23 | 0.806 |
| Body mass index, kg/m² | 24.36 ± 4.18 | 25.39 ± 5.13 | 24.15 ± 3.95 | 0.208 |
| Hypertension | 80 (63.49%) | 16 (72.73%) | 64 (61.54%) | 0.455 |
| Diabetes | 44 (34.92%) | 8 (36.36%) | 36 (34.62%) | 1.000 |
| Liver cirrhosis | 2 (1.59%) | 1 (4.55%) | 1 (0.96%) | 0.777 |
| Heart failure | 4 (3.17%) | 1 (4.55%) | 3 (2.88%) | 1.000 |
| Chronic kidney disease | 6 (4.76%) | 0 ( 0.0%) | 6 (5.77%) | 0.546 |
| Hospitalization, days | 6.34 ± 3.09 | 6.45 ± 2.82 | 6.32 ± 3.15 | 0.851 |
| Antibiotic start time, hours | 2.85 ± 1.25 | 2.77 ± 1.51 | 2.87 ± 1.19 | 0.753 |
| Hemoglobin, g/dL | 12.06 ± 1.72 | 12.43 ± 1.76 | 11.98 ± 1.71 | 0.269 |
| WBC counts, x10^3^/uL | 13.79 ± 5.44 | 14.47 ± 5.58 | 13.64 ± 5.43 | 0.520 |
| C-reactive protein, mg/dL | 17.48 ± 11.54 | 13.33 ± 6.19 | 18.40 ± 12.25 | 0.007 |
| Serum albumin, g/dL | 3.60 ± 0.46 | 3.73 ± 0.38 | 3.58 ± 0.47 | 0.164 |
| CT finding |  |  |  | 0.651 |
| Negative | 31 (24.60%) | 5 (22.73%) | 26 (25.00%) |  |
| Unilateral | 58 (46.03%) | 12 (54.55%) | 46 (44.23%) |  |
| Bilateral | 37 (29.37%) | 5 (22.73%) | 32 (30.77%) |  |
| Fever at 48 h | 37.35 ± 0.63 | 37.15 ± 0.50 | 37.39 ± 0.65 | 0.112 |
| Body temperature over 37.7℃ |  |  |  |  |
| Hospital hour: 24 | 48 (38.10%) | 10 (45.45%) | 38 (36.54%) | 0.589 |
| Hospital hour: 40 | 39 (31.20%) | 7 (31.82%) | 32 (31.07%) | 1.000 |
| Hospital hour: 48 | 30 (24.00%) | 3 (13.64%) | 27 (26.21%) | 0.328 |
| Hospital hour: 56 | 24 (19.20%) | 5 (22.73%) | 19 (18.45%) | 0.869 |
| Hospital hour: 64 | 17 (13.71%) | 4 (18.18%) | 13 (12.75%) | 0.741 |
| Hospital hour: 72 | 17 (13.93%) | 3 (13.64%) | 14 (14.00%) | 1.000 |

CT: computed tomography, FQ: fluoroquinolone, WBC: white blood cell
Continuous variables are presented as the mean ± standard deviation and categorical variables are presented as number (percentage).

**Supplementary Table 2. Logistic regression model assessing the link between body temperature over 37.4℃ at 48hrs and ESBL-producing bacteria**

|  | **Univariate** | | **Model 1** | | **Model 2** | |
| --- | --- | --- | --- | --- | --- | --- |
|  | **OR (95% CI)** | **p** | **OR (95% CI)** | **p** | **OR (95% CI)** | **p** |
| Over 37.4℃ at 48 h | 1.15 (1.05 - 1.26) | 0.004 | 1.14 (1.04 - 1.26) | 0.008 | 1.15 (1.04 - 1.26) | 0.006 |
| Sex (female) | 0.84 (0.74 - 0.95) | 0.006 | 0.83 (0.74 - 0.94) | 0.004 | 0.84 (0.74 - 0.95) | 0.005 |
| Age | 1.00 (1.00 - 1.00) | 0.476 | 1.00 (1.00 - 1.00) | 0.570 | 1.00 (1.00 - 1.00) | 0.683 |
| BMI | 0.99 (0.98 - 1.00) | 0.249 |  |  | 0.99 (0.98 - 1.00) | 0.235 |
| DM | 1.11 (1.00 - 1.23) | 0.046 | 1.11 (1.00 - 1.23) | 0.046 | 1.11 (1.00 - 1.23) | 0.041 |
| CKD | 1.07 (0.88 - 1.29) | 0.509 |  |  | 1.02 (0.84 - 1.24) | 0.821 |
| HTN | 0.95 (0.86 - 1.05) | 0.312 |  |  |  |  |
| LC | 0.89 (0.56 - 1.43) | 0.638 |  |  |  |  |
| HF | 1.10 (0.90 - 1.36) | 0.358 |  |  |  |  |
| Antibiotic start time | 0.96 (0.93 - 1.00) | 0.028 | 0.97 (0.93 - 1.00) | 0.061 | 0.97 (0.93 - 1.00) | 0.069 |
| Initial antibiotic |  |  |  |  |  |  |
| Cephalosporin |  |  |  |  |  |  |
| Fluoroquinolone | 1.02 (0.92 - 1.12) | 0.731 |  |  |  |  |
| P/β | 1.08 (0.75 - 1.55) | 0.682 |  |  |  |  |
| Others | 1.16 (0.59 - 2.27) | 0.667 |  |  |  |  |
| Hemoglobin | 0.99 (0.96 - 1.02) | 0.513 |  |  |  |  |
| WBC count | 1.00 (0.99 - 1.01) | 0.424 |  |  |  |  |
| CRP | 0.99 (0.99 - 1.00) | 0.023 | 0.99 (0.99 - 1.00) | 0.019 | 0.99 (0.99 - 1.00) | 0.022 |
| Albumin | 1.02 (0.91 - 1.14) | 0.713 |  |  |  |  |
| Model 1: sex, antibiotic start, CRP, DM, age | | | | | | |
| Model 2: sex, antibiotic start, CRP, DM, age, BMI, CKD, | | | | | | |
| BMI: body mass index, CKD: chronic kidney disease, CT: computed tomography, DM: diabetes mellitus, HF: heart failure, HTN: hypertension, LC: liver cirrhosis, P/β : penicillin/β-lactamase inhibitor, WBC: white blood cell | | | | | | |
| Logistic regression model for prolonged fever. Adjustable items were selected by univariate model and clinically importance. | | | | | | |

**Supplementary Table 3. Logistic regression model assessing the link between body temperature over 37.9℃ at 48hrs and ESBL-producing bacteria**

|  | **Univariate** | | **Model 1** | | **Model 2** | |
| --- | --- | --- | --- | --- | --- | --- |
|  | **OR (95% CI)** | **p** | **OR (95% CI)** | **p** | **OR (95% CI)** | **p** |
| Over 37.9℃ at 48 h | 1.19 (1.06 - 1.33) | 0.003 | 1.19 (1.06 - 1.33) | 0.004 | 1.20 (1.06 - 1.35) | 0.003 |
| Sex (female) | 0.84 (0.74 - 0.95) | 0.006 | 0.84 (0.74 - 0.95) | 0.005 | 0.84 (0.74 - 0.95) | 0.007 |
| Age | 1.00 (1.00 - 1.00) | 0.476 | 1.00 (1.00 - 1.00) | 0.418 | 1.00 (1.00 - 1.00) | 0.511 |
| BMI | 0.99 (0.98 - 1.00) | 0.249 |  |  | 0.99 (0.98 - 1.00) | 0.210 |
| DM | 1.11 (1.00 - 1.23) | 0.046 | 1.10 (0.99 - 1.22) | 0.064 | 1.10 (1.00 - 1.22) | 0.059 |
| CKD | 1.07 (0.88 - 1.29) | 0.509 |  |  | 1.04 (0.86 - 1.27) | 0.665 |
| HTN | 0.95 (0.86 - 1.05) | 0.312 |  |  |  |  |
| LC | 0.89 (0.56 - 1.43) | 0.638 |  |  |  |  |
| HF | 1.10 (0.90 - 1.36) | 0.358 |  |  |  |  |
| Antibiotic start time | 0.96 (0.93 - 1.00) | 0.028 | 0.96 (0.93 - 1.00) | 0.042 | 0.96 (0.93 - 1.00) | 0.049 |
| Initial antibiotic |  |  |  |  |  |  |
| Cephalosporin |  |  |  |  |  |  |
| Fluoroquinolone | 1.02 (0.92 - 1.12) | 0.731 |  |  |  |  |
| P/β | 1.08 (0.75 - 1.55) | 0.682 |  |  |  |  |
| Others | 1.16 (0.59 - 2.27) | 0.667 |  |  |  |  |
| Hemoglobin | 0.99 (0.96 - 1.02) | 0.513 |  |  |  |  |
| WBC count | 1.00 (0.99 - 1.01) | 0.424 |  |  |  |  |
| CRP | 0.99 (0.99 - 1.00) | 0.023 | 0.99 (0.99 - 1.00) | 0.016 | 0.99 (0.99 - 1.00) | 0.020 |
| Albumin | 1.02 (0.91 - 1.14) | 0.713 |  |  |  |  |
| Model 1: sex, antibiotic start, CRP, DM, age | | | | | | |
| Model 2: sex, antibiotic start, CRP, DM, age, BMI, CKD, | | | | | | |
| BMI: body mass index, CKD: chronic kidney disease, CT: computed tomography, DM: diabetes mellitus, HF: heart failure, HTN: hypertension, LC: liver cirrhosis, P/β : penicillin/β-lactamase inhibitor, WBC: white blood cell | | | | | | |
| Logistic regression model for prolonged fever. Adjustable items were selected by univariate model and clinically importance. | | | | | | |

**Supplementary Figure 1. Restricted odds ratio spline curve for body temperature over 37.7℃ at 48hr**

This graph showed that Odds ratio increased over body temperature of 37.7℃ at 48hrs (blue line).

**
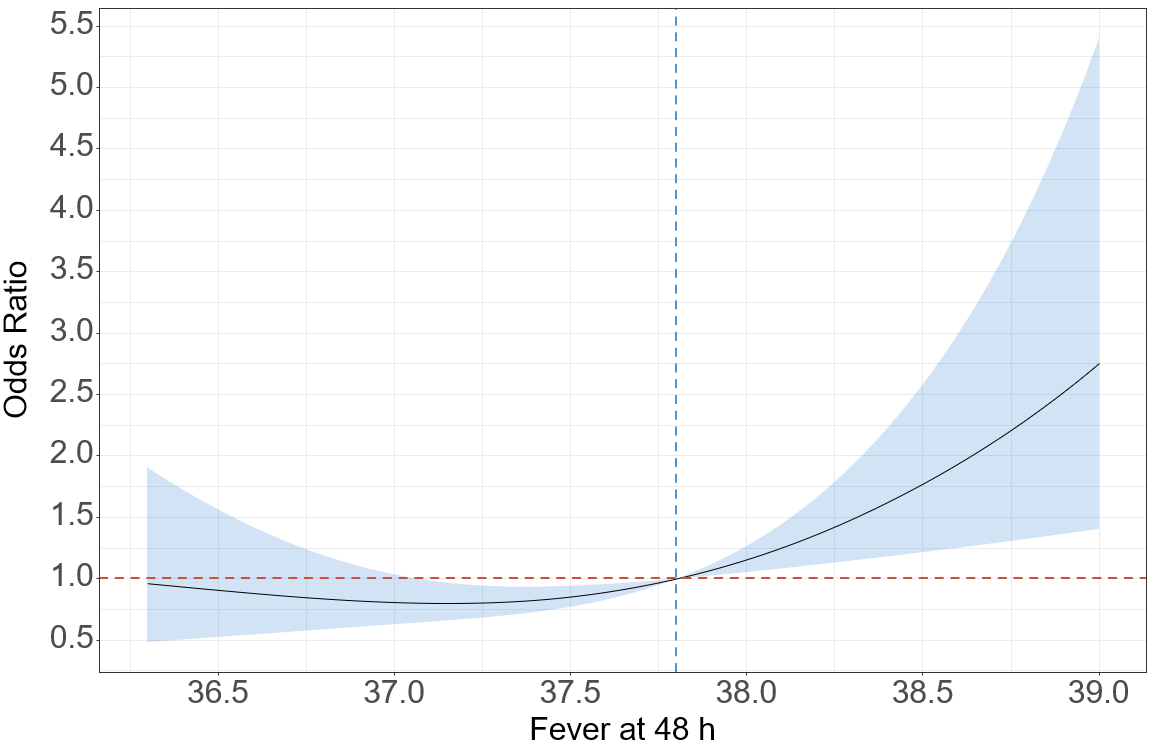
**
